# Adherence to a six-dose regimen of artemether-lumefantrine among uncomplicated *Plasmodium falciparum *patients in the Tigray Region, Ethiopia

**DOI:** 10.1186/1475-2875-10-349

**Published:** 2011-12-05

**Authors:** Hailemariam Lemma, Curt Löfgren, Miguel San Sebastian

**Affiliations:** 1 Tigray Health Bureau, P.O. Box 7, Mekelle, Ethiopia; 2 Epidemiology and Global Health, Umeå University, SE-901 85, Umeå, Sweden

## Abstract

**Background:**

In 2004, Ethiopia switched its first-line treatment of uncomplicated *Plasmodium falciparum *malaria from sulphadoxine-pyrimethamine to a fixed artemisinin-based combination therapy (ACT), artemether-lumefantrine (AL). Patient adherence to AL regimen is a major determining factor to achieve the desired therapeutic outcome. The aim of this study was to measure patient adherence levels to the six-dose AL regimen for the treatment of uncomplicated *P. falciparum *malaria and to identify its determinant factors in rural areas of the Tigray region, Ethiopia

**Methods:**

The study was conducted under routine health service delivery at health posts level. Patients/caregivers were not informed about their home visit and were traced on the day after they finished the AL regimen. By combining the response to a structured questionnaire and the tablet count from the blister, adherence level was classified into three categories: definitely non-adherent, probably non-adherent and probably adherent. Reasons for being definitely non-adherent were also assessed. For the purpose of examine risk factors, definitely non-adherent and probably non-adherent was merged into a non-adherent group. Variables found significantly associated (*p *< 0.05) with the adherence level on the univariate analysis were fitted into a multivariate logistic regression model.

**Results:**

Out of the total initially enrolled 180 patients, 86.1% completed the follow-up. Out of these, 38.7% were classified as probably adherent, 34.8% as probably non-adherent, and 26.5% were definitely non-adherent. The most common reasons that definitely non-adherents gave for not taking the full dose were "too many tablets" (37.3%) and to "felt better before finished the treatment course" (25.5%). The adherence of the patients was associated with the ownership of a radio (adjusted odd ratio, AOR: 3.8; 95% CI: 1.66-8.75), the belief that malaria can be treated traditionally (AOR: 0.09; 95% CI: 0.01-0.78) and a delay of more than one day in seeking treatment after the onset of fever (AOR: 5.39; 95% CI: 1.83-15.88).

**Conclusion:**

The very low adherence to AL found in this study raises serious concerns for the malaria control in the region. The implementation of a monitoring adherence system is essential to ensure long-term treatment efficacy.

## Background

Despite the current surge of global efforts to scale up malaria control interventions, the disease is still the leading cause of morbidity, mortality and economic losses in sub-Saharan African (SSA) countries [1,2]. Ethiopia is among the countries which contribute most to this burden. It is estimated that three quarters of the country's total area is malarious and an approximated 54 million (68%) of the total population of 80 million (projected from 2007 census for 2010) live in areas where they are at risk for the disease [3,4]. Unlike most parts of SSA where most, if not all, malaria infections are attributed to *Plasmodium falciparum*, the parasite pool in Ethiopia is co-dominated by both *P. falciparum *and *Plasmodium vivax*, sharing approximately 60% and 40% of the cases, respectively [3,5].

In fighting this deadly disease, provision of early diagnosis and prompt and effective treatment is the core of malaria control strategies in the country [5]. However, wide emergence of drug resistance to *P. falciparum *has been one of the main barriers to the effectiveness of this strategy. As result of the 2003 nationwide anti-malarial *in vivo *therapeutic efficacy assessment, the country switched its first-line treatment of uncomplicated *P. falciparum *malaria from sulphadoxine-pyrimethamine (SP) to a fixed artemisinin-based combination therapy (ACT), artemether-lumefantrine (AL).

Clinical trials have shown that AL is a highly efficacious and safe anti-malarial drug [6-11]. However, several issues need to be addressed in order to achieve its desired therapeutic outcome [12]. Among many, patient adherence to the treatment regimen is a major determining factor [13,14]. Poor adherence results in sub-therapeutic drug concentrations which fail to provide a successful cure. Furthermore, the presence of sub-curative concentrations of the anti-malarial drug in the blood will only eliminate the most sensitive parasites, allowing those that are less sensitive to survive [15]. This contributes to the spread of drug resistant mutant strains of the malaria parasite [16-18], thus complicating the treatment scenario.

The need to take unsupervised AL twice daily for three successive days at the right dose and correct time interval is challenging and might increase the risk for poor adherence. Thus, understanding the patient adherence level to this six-dose regimen of AL for the treatment of *P. falciparum *and identifying its possible determinant factors is crucial in the provision of effective malaria treatment. However, despite the fact that AL is the most widely deployed anti-malarial [1,19], studies on this treatment are limited, and even the available studies not only demonstrate different levels of adherence, but also account for different risk factors[20-23]. While high levels of adherence (74-98%) have been found in most African studies [2,22,24,25], lower levels have been reported in a study from southern Sudan (40%) [20]. A literature review on adherence to various types of anti-malarial drugs identified different definitions, methods, criteria and results. The review also revealed that the quality and quantity of information were inadequate and the results varied depending on the local setting [23]. These variations highlight the need for local evidence, which is practically lacking in the Ethiopian context.

The aim of this study was to measure patient adherence levels to the six-dose AL regimen for the treatment of uncomplicated *P. falciparum *and to identify its determinant factors in rural areas of the Tigray region, Ethiopia. The results from this research will help to develop and implement effective communication tools prompting an effective diagnosis and treatment strategy.

## Methods

### Study area

Tigray is the northernmost regional state of Ethiopia. The region has approximately 4.6 million inhabitants (projected from the 2007 census for 2010); most of them (80.5%) live in rural areas and depend on subsistence agriculture [4]. As in the rest of Ethiopia, malaria transmission in Tigray is very seasonal, unstable and occurs mainly at altitudes below 2,000 m above sea level (masl). Around 65% of the population in Tigray is at risk of malaria and the disease is a leading public health problem in the region [26,27].

The region has a four-tier health delivery system with the primary health care units (PHCU) at the grass-roots level. A PHCU includes a health centre and five satellite health posts [4]. A health post, planned to serve an average of 5,000 inhabitants, is staffed by two health extension workers (HEWs). HEWs are high school graduates with 1 year of training on community-based heath programmes, including malaria diagnosis and treatment. At this level, malaria confirmatory diagnosis is done using rapid diagnostic tests (RDTs) while maintaining a clinical presumptive approach if an RDT is not available. AL is the drug of choice for treating *P. falciparum *and chloroquine is used for non-*P. falciparum *malaria cases. Malaria diagnosis and treatment is provided free of charge [28,29].

### Study design and subjects

This study was conducted in three randomly-selected malarious districts: Raya-azebo and Mereb-leke (1,500-2,000 masl) and Tahty-adiabo (< 1,000 masl). In all the three districts, approximately one third of the cases in the outpatient service were due to malaria [27].

The study was conducted under routine health service delivery at health posts level in 2008 in the period of August-November, the peak malaria transmission season. Participants were patients who sought treatment from the HEW at the health post or village. They were included if they were: i) residents of the catchment area of the health post; ii) positive for the Paracheck Pf test (*P. falciparum*-specific RDT device); iii) older than 2 months of age and iv) patients treated before noon. Patients receiving the first dose in the afternoon would require the second and some other doses at night. Due to the difficulty of relating these doses time with a natural event, those cases were excluded Patients were excluded if: i) they exhibited signs and symptoms of sever disease; ii) there was already a household member enrolled in the study (no family was interviewed twice); iii) they were pregnant mothers in their first trimester and iv)they had taken AL within the past 2 weeks.

The number of patients recruited each day by an enumerator was limited to a maximum of three as tracing of participants at their homes was difficult and time consuming. When the number of patients meeting the inclusion criteria in 1 day exceeded three, they were randomly selected. In the situation where more than one patient in a day from the same household was treated, the younger patient was included in order to enrol as many children as adults. If both were adults, one of them was randomly selected. Patients/caregivers were not informed about the visit to their home.

Sample size was calculated based on the assumption of 25% non-adherence with a precision of 10% and a design effect of 2 at a 95% confidence interval (CI). After accounting for the 20% drop-out rate (including non-replaced immediate spat and/or vomited dose), a total sample size of 175 participants was required.

### Patient management and dose instruction

The AL tablet (Coartem^®^, Novartis Pharma AG, Basel, Switzerland) is presented in an illustrated patient-friendly blister pack according to four age groups. The blister is divided into six compartments, one for each dose. AL is administered: (i) one tablet (artemether 20 mg/lumefantrine 120 mg) per dose for children > 2 months to 2 years of age; (ii) two tablets per dose for children three to seven years of age; (iii) three tablets per dose for children eight to 10 years of age and; (iv) four tablets per dose for those over 10 years of age [5].

Eligible patients were given the first dose under supervision and observed for 30 min. If vomiting occurred within this period, the dose was repeated and if vomiting persisted, patients were excluded from the study and referred to the next higher health facility. Instructions on dosing and frequency/time interval of the remaining five doses were given to patients/caregivers. The instruction aimed to achieve the second dose to be taken 8 hours after the first dose and the remaining four doses at a 12-h interval over the next 2 days. Patient/caregivers were instructed that if a dose was spat out or vomited within half an hour after dosing (vomit contains the drug suspension), a full dose should be re-administered with the possibility of refilling/replacing it from the health post. They were also told that all doses should preferably be taken with fat-containing food, such as milk and nuts. In the case of small children, caregivers were told to dissolve the tablets in water. They were also advised to visit the next higher health facility if they felt worse or showed no improvement.

### Data collection and patient tracing

A structured questionnaire was developed based on Fogg et al. and Depoortere et al. [30,31], translated to the local language and administered in the form of an interview. Enumerators (HEWs) were trained on: inclusion and exclusion criteria and the use of the questionnaire using mock exercises applying different scenarios. On day zero, the enrolment day, baseline data including socio-demographic characteristics, chief complaints, history of prior medication for the current illness, residence village (tracing address) and current prescription was collected. Patients were traced at their home on the day after they were supposed to have finished the course of AL (day 3). The first task of the enumerator was to check the availability of the blister pack and to inspect for remaining tablets. Then, day-by-day information on the number of doses, number of tablets in each dose, time of each dose, reasons for any leftover or missed dose and the presence or absence of vomiting was collected. If vomiting was present, the estimated time after intake and action taken was recorded. Reasons for any leftover or missed dose were also gathered. For patients under 15 years old, respondents were their parents/caregivers. Patients/caregivers who were not available in their home on day 3 were again traced on day four and if not found, they were considered as lost to follow-up. During the tracing day, if a patient was found still to be sick, (s)he was immediately referred to the closest higher-level health facility.

### Definition of adherence and inter-dose interval

By combining the response to the oral interview and the tablet count from the blister pack, adherence level was classified into three categories: definitely non-adherent, probably non-adherent and probably adherent [22,31]. A patient who had leftover tablet(s) in a blister pack was straightforwardly classified as definitely non-adherent (DNA). When the blister pack was either missing or empty, but the patient did not report either taking all doses at the given time interval (on the correct day or correct timing) or at the correct amount was classified as probably non-adherent (PNA). Probably adherent (PA) was a patient who reported taking all doses, at the given time interval (on the correct day and correct timing), at the correct amount, and with no spitting or vomiting within the first 30 min or when such spat/vomited dose was re-administered.

Patients who did not re-administer any spat or vomited dose which occurred within the first 30 min were excluded from the analysis. The timing dose given by the respondent was in association with a natural event, such as the position of the sun, coffee time, cow milking, time from church and time of cattle leaving or entering their shed. Converting theses events to approximate hours required flexibility. Therefore, the time interval for the second dose was considered correct if taken between 8-10 h after the first dose while subsequent doses were in the interval of 12 h with a range of plus/minus 2 h from the preceding dose.

### Data management and analysis

All collected data were entered and cleaned with Epi-Info version 3.4.2 (CDC, Atlanta, GA, USA) and then analysed using Stata 10 (Stata Corp., College Station, TX, USA) software.

The three levels of adherence were calculated and presented as proportions. Risk factors of adherence were examined. For this purpose, definitely non-adherent and probably non-adherent were merged into a non-adherent group, transforming the outcome variable into binary. The association between adherence level (the dependent variable) and several exposure variables (sex, age group, highest education level in the family, presence/absence of a radio), history of prior medication (traditional and/or modern), presence/absence of easily noticeable symptoms (fever, shiver, vomiting, jaundice), health improvement after starting treatment, time lag between the onset of fever and treatment, family size, presence of a volunteer community health worker (VCHW) in the family and belief that traditional medicine cures malaria) was first analysed. In a second step, variables significantly associated (*p *< 0.05) with the adherence level were fitted into a multivariate logistic regression model.

### Ethical considerations

The study was approved by the Tigray Health Bureau. On the day of follow-up (tracing), patients were informed about the purpose of the study, and participants or caregivers (for patients less than 18 years old) were asked to provide verbal consent as majority of the participants were illiterate.

## Results

### Patient/guardian general characteristics

**Figure 1:**
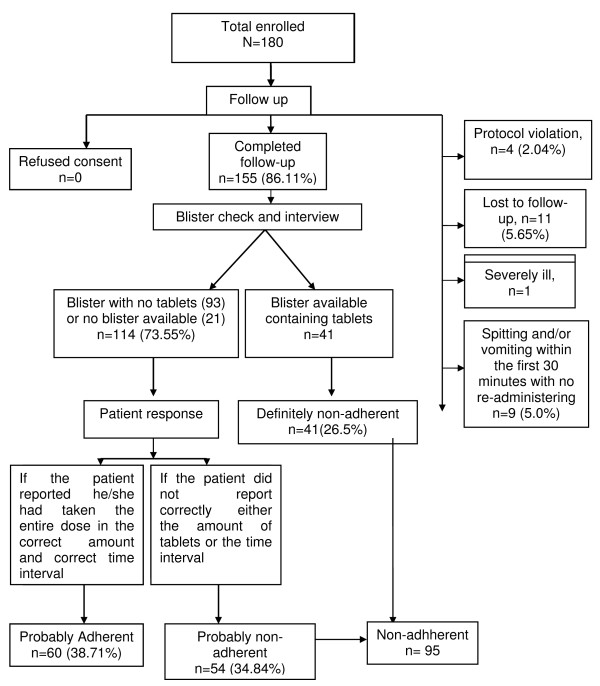
**Flow diagram showing enrollment, follow-up and adherence level**.

Out of the total initially enrolled 180 patients, 86.1% (n = 155) completed the follow-up. None of the patients refused to be involved in the study. Lost to follow-up (5.6%) was the major reason for exclusion from the analysis followed by spitting/vomiting a dose within 30 min with no replacement (5.0%), protocol violation (2.0%) and severity of illness (one case) (Figure 1). The protocol violation occurred in young children (< 11 years old) and all the lost to follow-up cases were adults aged > 20 years old.

For the patients who completed the follow-up, the baseline characteristics are shown in Table 1. The majority (66.5%) were above 10 years old. Nearly all the patients (93.0%) had a history of fever and 31.6% had an axillary temperature > 38.5°C on the day of diagnosis. Since neither a child him/herself nor the caregiver on his/her behalf could accurately express some of the disease symptoms, headache, backache, chills/rigors and joint pain were excluded from the analysis. Symptoms included were shivering (47.7%), vomiting (43.9%) and jaundice (5.21%). Around one third of the patients sought treatment within one to two days (26.4 and 27.1%, respectively) after recognising the symptoms. Patients reporting prior uptake of either modern medication (other than AL) or traditional medicine for the current episodes were 6.5% and 2%, respectively. Eighteen (12.8%) patients believed that malaria could be treated traditionally.

**Table 1:** Patients' characteristic by adherence level to AL, Tigray, Ethiopia 2008

Patients/guardian characteristic (n = 155)	n (%)	DNA n = 41 (%)	PNA n = 54 (%)	PA n = 60 (%)
Sex				

Male	74 (47.7)	24 (58.5)	23 (42.6)	27 (45.0)

Female	81 (52.3)	17(41.5)	31 (57.4)	33 (55.0)

Treatment age group				

3 months-2 years	7 (4.5)	4 (9.8)	1 (1.9)	2 (3.3)

3-7 years	28 (18.1)	10 (24.4)	5 (9.3)	13 (21.7)

8-10 years	17 (11.0)	5 (12.2)	9 (16.7)	3 (5.0)

> 10 years	103 (66.5)	22 (53.6)	39 (72.0)	42 (70.0)

Age group				

≤10 years	52 (33.5)	19 (46.3)	15 (27.8)	18 (30.0)

11-14 years	23 (14.8)	9 (22.0)	4 (7.4)	10 (16.7)

≥15 years	80 (51.6)	13 (31.7)	35 (64.8)	32 (53.3)

Chief complaint (Yes)*				

Fever	144 (92.9)	40 (97.6)	48 (88.9)	56 (93.3)

Shiver	74 (47.7)	20 (48.8)	24 (44.4)	30 (50.0)

Vomit	68 (43.9)	22 (53.7)	16 (29.6)	30 (50.0)

Jaundice	8 (5.2)	2 (4.9)	1 (1.8)	5 (8.3)

Axillary temperature (°C) at day zero				

= < 37.5	24 (15.5)	6 (14.6)	10 (18.5)	8 (13.3)

37.6-38.5	82 (52.9)	22 (53.7)	30 (55.6)	30 (50.0)

38.6-39.5	49 (31.6)	13 (31.7)	14 (25.9)	22 (36.7)

VCHW in family (Yes)	10 (6.5)	1 (2.4)	5 (9.2)	4 (6.7)

Possession of radio (Yes)	81 (52.3)	8 (19.5)	27 (50.0)	46 (76.7)

Education of patient/caregiver (Illiterate)	127 (81.9)	38 (92.7)	47 (87.0)	42 (70.0)

Prior modern medicine (Yes)	10 (6.5)	4 (9.8)	3 (5.0)	3 (5.6)

Prior traditional medicine (Yes)	3 (1.9)	2 (4.9)	1 (1.9)	0 (0.0)

Belief that malaria treated traditionally (Yes)	18 (12.8)	7 (19.4)	10 (19.6)	1 (1.9)

Response to current treatment (Improved)	148 (95.5)	34 (23.0)	54 (36.5)	60 (40.5)

Days between onset of fever and treatment				

1 day	41 (26.4)	9 (22.0)	25 (46.3)	7 (11.7)

2 day	42 (27.1)	7 (17.0)	15 (27.8)	20 (33.3)

3-5 days	72 (46.5)	25 (61.0)	14 (25.9)	33 (55.0)

Family size				

1-3	29 (18.7)	9 (22.0)	8 (14.9)	12 (20.0)

4-6	70 (45.2)	19 (46.3)	24 (44.4)	27 (45.0)

> 6	56 (36.1)	13 (31.7)	22 (40.7)	21 (35.0)

Number of children under 10 years**				

0	30 (19.7)	9 (22.0)	8 (15.0)	13 (22.4)

1-2	80 (52.6)	17 (41.5)	28 (52.8)	35 (60.3)

> = 3	42 (27.6)	15 (36.6)	17 (32.0)	10 (17.2)

* Row-wise as column-wise presentation is less informative. The figures represent those who reported the particular symptom out of the total cases (N = 155).

**152 families

In most households (n = 127, 81.9%), either the patient or his/her parents were illiterate or below grade 5 with only few (n = 24) who had attended medium school (grades 5-8) with remaining attended high school. Almost half (45.2%) of the households had a family size ranging from four to six members, 27.1% had three or more children < 10 years old, 6.5% had a VCHW family member and 52.3% owned a radio. Ninety-six percent felt better in response to the current treatment.

### Adherence level

Out of the total follow-up participants, 94.2% were traced on day 3 and the rest on day 4. Nearly three quarters of the patients (73.5%) reported to have completed the treatment. Out of these, 54 did not correctly report the dose or the time interval and were classified as probably non-adherent; the rest (n = 60) were classified as probably adherent (Figure 1). Errors in frequency (timing of dose) accounted for almost three quarters of the probably non-adherent group. Forty-one patients (26.5%) were found with tablets in the blister and were thus classified as definitely non-adherent.

In order to assess the age adherence pattern, we classified the subjects into three age groups based on their ability to make decisions on taking the drug: under 10 years old (completely dependent on their parents), 10-15 years old (partially dependent) and above 15 years old (independent of their parents). The oldest age group was mainly PA (40.0%) and PNA (43.8%) while the 10-15 year old group was overall PA (43.5%) and DNA (39.1%). The youngest age group was fairly equally distributed among the three categories.

Definitely non-adherents gave one or more reasons for not taking any or all of the doses. The most common reasons were "too many tablets" (37.3%) and to "felt better before finished the treatment course" (25.5%). Refusal to take the tablets (7.8%) and "tablets too big to swallow" (3.9%) were also mentioned as explanations for not taking the tablets. Other less frequent reasons were "tablets bitter", "forgot", "no improvement" and "kept for future episodes". Only one patient/caregiver (2.4%) claimed not to understand the instructions (Table 2). Six patients neither reported finishing the doses nor showed leftover tablets. When asked, the only reasons they gave were that they shared with others (n = 4) or kept the medication for future episodes (n = 2).

**Table 2:** Reasons among definitely non-adherent patients (n = 41) for interrupting the treatment

Why tablet leftover	n (%)
Too many tablet)	19 (37.3)

Felt better before treatment course finished	13 (25.5)

Tablet was bitter or forgot or no improvement or keep for future	12 (23.5)

Refused	4 (7.8)

Tablets too big	2 (3.9)

Don't understand the instruction	1 (2.0)

Of the patients who reported immediate spitting or vomiting within 30 min (n = 14), only five cases re-administered the dose; two of them borrowed from their neighbour and the other three shifted the doses. Despite the instruction given, no patients replaced the tablets from the health post.

Four variables were found to be significantly associated with being adherent in the univariate analysis: the ownership of a radio, the belief that malaria cannot be treated traditionally, a delay of more than 1 day in seeking treatment after the onset of fever and literacy. When fitted into the multivariate logistic regression model, the ownership of a radio (adjusted odd ratio, AOR: 3.8; 95% CI: 1.66-8.75), the belief that malaria can be treated traditionally (AOR: 0.09; 95% CI: 0.01-0.78) and a delay of more than 1 day in seeking treatment after the onset of fever (AOR: 5.39; 95% CI: 1.83-15.88) remained significant (Table 3).

**Table 3:** Factors associated with adherence level to AL: univariate and multivariate logistic regression model, Tigray, 2008

Patient/guardian characteristic	Adherent		Non-adherent		Odd ratio(OR) (95%CI)	Adjusted (AOR) (95%CI)
	**n**	**(%)**	**n**	**%**		

Sex						

Female	27	36.49	47	63.51	1	-

Male	33	40.74	48	59.26	1.12 (0.44-1.60)	

Age group						

10 years	18	34.62	34	65.38	1	

10-15	10	43.48	13	56.52	1.45 (0.53-3.96)	-

> = 15 years	32	41.00	48	60.00	1.26 (0.61-2.60)	-

Chief complaints						

Fever						

No	4	36.36	7	63.64	1	-

Yes	56	38.89	88	61.11	1.11 (0.31-3.98)	

Shivering						

No	30	37.04	51	62.96	1	

Yes	30	40.54	44	59.46	1.16 (0.61-2.21)	-

Vomiting						

No	30	34.48	57	65.52	1	-

Yes	30	44.12	38	55.88	1.5 (0.78-2.88)	

Jaundice						

No	55	37.41	92	62.59	1	

Yes	5	62.50	3	37.50	2.79 (0.64-12.12)	-

Axillary temperature (°C) at day zero						

≤37.5	8	33.33	16	66.67	1	

≥ 37.6 and ≤ 38.4	30	36.59	52	63.41	1.15 (0.44-3.01)	-

≥ 38.5 and ≤ 39.5	22	44.90	27	55.10	1.63 (0.59-4.51)	-

VCHW in family						

No	56	38.62	89	61.38	1	-

Yes	4	40.00	6	60.00	1.06 (0.29-3.92)	

Possession of radio						

No	14	18.92	60	81.08	1	-

Yes	46	56.79	35	43.21	5.63 (2.72-11.68)	3.82 (1.66-8.75)

Education of patient/caregiver						

Illiterate	18	31.58	85	68.42	1	

Literate	42	34.29	10	65.71	1.83 (1.15-2.92)	2.27 (0.86-6.01)

Prior modern medicine						

No	57	39.31	88	60.69	1	

Yes	3	30.00	7	70..00	0.66 (0.18-1.54)	.-

Prior traditional medicine						

No	60	39.74	92	60.26	-	-

Yes	0	0.00	3	100.00	Dropped	-

Belief that malaria treated traditionally						

No	59	43.09	78	56.91	1	

Yes	1	5.56	17	94.44	0.08(0.01-0.60)	0.09 (0.01-0.78)

Response to current treatment						

Not improved	0	0	7	100	1	

improved	60	40.54	88	59.46	1.15 (0.55-2.40)	-

Days between onset of fever and treatment						

1 day	7	17.07	34	82.93	1	

2 day	20	47.62	22	52.38	4.42 (1.60-12-17)	5.03 (1.54-16.32)

3-5 days	33	45.83	39	54.17	4.11 (1.61-10.48	5.40 (1.83-15.88)

Family size						

1-3	12	41.38	17	58.62	1	

4-6	27	38.57	43	61.43	0.89 (0.37-2.15)	-

> 6	21	37.50	35	62.50	0.85 (0.34-2.12)	-

Number of children under 10 years old*						

0	13	43.33	17	56.67	1	-

1-2	35	43.75	45	56.25	1.02 (0.44-2.37)	-

> = 3	10	23.81	32	76.19	0.41 (0.15-1.13)	-

Zone/transmission intensity						

Below 1000 masl					1	

Between 1000-15000 masl					0.61 (0.32-1.17)	-

## Discussion

Adherence to AL is a capital issue in achieving effective implementation of the malaria case management strategy. The poor results obtained in this study population (26.5% were definitely non-adherers) raises great concern. Taking into consideration the probable non-adherent patients, the level of non-adherence could increase up to 61.3%. Few studies, mostly from African countries, have specifically assessed the level of adherence to the six-dose regimen of AL. The level of adherence in these studies ranged between 74% in Ghana to 98.3% in Tanzania [2,22,24,25,32]. However, low levels of adherence to AL similar to our study (40%) have also been reported in children aged less than 5 years in southern Sudan[20]. The only non-African study assessing adherence to AL comes from Bangladesh. This randomized controlled trial comparing directly observed vs. non-directly observed treatment (NDOT) obtained 93% adherence rate in the NDOT group [33]. While the enormous gap between these studies and ours could be real, several issues need to be taken into account that might partly explain these discrepancies.

One first issue relates to the type of context where the studies were carried out. For instance, in the Ugandan study [22], the sample was an educated semi-urban population living in a high endemic malaria area, whereas our study was placed in a rural setting with a predominantly illiterate population living in a low transmission setting where cases were managed at rural health facilities. Populations in high endemic areas are more aware of malaria and its consequences [34], and semi-urban populations are also more likely to have better access to health information than in rural settlements

A second issue to consider is the design of the study. In a study conducted in Tanzania, a level of adherence to AL of approximately 90% was reported. However, patients or caregivers were informed that there would be a follow-up visit, which could influence patient/caregiver behaviour. In another study from Ghana, patient follow-up took place between 4 and 14 days after the initial dose. In such cases, blister pack inspection for leftover tablets with this lag of time might not show real adherence. In addition, the close supervision carried out by the research team could have contributed to the high level of compliance reported [24].

A third aspect relates to the definition of adherence. In this study, a very strict definition was used, including patients who reported taking all doses at the given time interval (on the correct day and correct timing), at the correct amount and with no spitting or vomiting within the first 30 min, or such spat/vomited dose was re-administered. In some previous studies, the inter-dose time interval was either not clearly described [22,25,32,33] or very broadly defined [2]. For instance, in the study from Ghana (adherence level 92.5%), adherence was based on the description of how AL was given by the caregivers but no specific information about the time interval or the correct dose seems to have been collected [24]. In the study from Zambia, the inter-dose timing (+/-4 h) was wide compared to this one (+/-2 h), thus increasing the number of probably adherent patients [2]. In fact, three fourths of the probably non-adherent classifications in our study were due to incorrect timing.

Several reasons were presented by patients/caregivers on why they did not finish the treatment course. The most common reason was "too many tablets" (37.3%). This concern was mainly expressed by caregivers of children less than 15 years old. Parents/caregivers might be fearful to give many tablets to their small child. Thus, they might have modified either the dosing or timing or both and were ultimately found to be non-adherent. The rapid fever clearance and clinical recovery under AL treatment seemed to encourage patients to give up the regimen. "Felt better before finishing the regimen" was the second reason (25.5%) for not taking all the tablets. Different studies have also pointed to an improvement in the condition as the main reason for treatment discontinuation [14,22,24,33]. Reasons such as "refused to take", "bitter", and "too big to swallow" were restricted to children aged less than 5 years. This indicated that the AL tablets were inconvenient for caregivers to administer. The current introduction of a dispersible paediatric formulation of AL might contribute to overcoming this problem [35].

One commonly reported reason for lack of adherence in many other studies was misunderstanding the instructions provided by the health care personnel [31,36]. In the present study, this was low (2.4%). One potential explanation of this finding might be that the HEWs were native residents from the area, which could increase the communication confidence of the patient. In addition, the pictogram and clustering of the doses within the blister packet might have contributed to understand dosing and inter-dose spacing. On the other hand, given that the HEWs were in this study providers and data collectors at the same time, patients might have been reluctant to report misunderstandings. Although side-effects have been reported with other drugs as a reason for not completing the treatment course [37], this was not the case with AL in the current study.

Three risk factors were strongly associated with being adherent: owning a radio, the belief that malaria cannot be treated traditionally and a delay in treatment seeking. The Tigray Health Bureau has made an effort to communicate health messages on malaria prevention and control strategies via posters, leaflets, community gatherings and radio messages. Radio broadcasting health messages is a well-known health promotion tool. A previous study in the area has shown that the ownership of a radio also increases the knowledge about malaria and the practice of prevention measures, such as the use of long-lasting insecticidal nets [34]. Those who sought treatment more than 1 day after the onset of fever were 5.4 times more likely to adhere to the treatment regimen than those who sought treatment earlier. This could be because the more a patient delays the visit, the more they suffer and the likelihood of treatment-seeking increases with the severity of symptoms [14]. The delay in treatment-seeking as promoting factor for adherence should be interpreted with some caution. Though it increased adherence, this should be discouraged since it contradicts with the national and regional goal of 100% of suspected malaria cases diagnosed and treated within 24 h of the onset of illness [3]. The belief that malaria can be treated traditionally supported the possibility of interrupting AL treatment in favour of traditional medicine. Education has been found to be an important risk factor for adherence in some studies [22,31,33]. However, in agreement with research from Bangladesh, education did not play any role in this study. A probable explanation would be the high proportion of uneducated people in this rural area.

This study has also identified some issues of concern. First, although few, patients reported borrowing/sharing tablets for replacing vomited doses or keeping them for future episodes. Second, protocol violations (prescription did not match the subject's age), which was seen only in children, indicated that the fear of "too many tablets" occurred not only among caregivers, but also among care providers. Third, despite the recommendation for early diagnosis and prompt treatment and improved formal health care access as the result of the HEP, only one-quarter of the patients sought treatment within 1 day of the onset of fever.

### Methodological considerations

Certain factors must be considered when interpreting these results. First, the self-reported method of adherence assessment is open to different biases such as recall and good-will bias. Particularly the latter can lead to a lower estimation of the true level of non-adherence as patients would be more likely to be unwilling to report missed doses [6,13,20]. The enumerators might also be resistant to collect negative responses as poor results might indicate their performance. However, the low level of adherence in our study seems not to reflect these concerns. Another limitation was that only reasons for definitely non-adherent (patients with leftover tablets) were assessed, but not reasons for being probably non-adherent and probably adherent. Furthermore, given the poor and rural context of our study, information on dose timing could not be collected accurately, which could have influenced the classification of patients.

## Conclusions

Adherence to recommended malaria drug regimens is a key determinant of the success of any malaria control programme. The current study in Tigray has shown very low adherence to AL. This brought about three serious concerns: i) the inadequate cure of the patient, ii) the risk of transmission due to parasite survivors in non-adherent patients and iii) the development of parasite resistance. This study has identified some aspects which the malaria control programme in Tigray should concentrate on. The use of radios should be promoted and radio-health messages regarding the importance of adherence to AL should be intensified. Specific training on communicating treatment instructions should be carried out with the HEWs. The implementation of a monitoring adherence system is essential to ensure long-term treatment efficacy.

## Competing interests

There is no conflict of interest and no business relationship with the manufacturer producing any of the anti-malarial drugs featured in this study

## Authors' contributions

HL developed the study design, collected and analyzed the data and drafted the manuscript. MSS, CL contributed to the study design and critically read and improved the manuscript. All authors read and approved the final manuscript.
